# The Columbus Knee System: 4-Year Results of a New Deep Flexion Design Compared to the NexGen Full Flex Implant

**DOI:** 10.1155/2012/213817

**Published:** 2012-03-07

**Authors:** D. Goebel, W. Schultz

**Affiliations:** ^1^Orthopaedic Clinic Donaueschingen, Karlstra*β*e 10, 78 166 Donaueschingen, Germany; ^2^Orthopaedic Department, University of Göttingen, Robert Koch Stra*β*e 40, 37 075 Göttingen, Germany

## Abstract

The Columbus knee system is designed as a standard knee implant to allow high flexion without additional bone resection. Between August, 2004 and March, 2010 we performed 109 total knee arthroplasties of the Columbus knee system in 101 consecutive patients suffering from primary arthrosis of the knee. Mean age was 72.4 years in women and 70.3 years in men. Mean followup was 47.3 months. The 4-year results of a group of patients who received the NexGen Full Flex implant operated by the same surgeon were used for comparison. Mean total knee score was Columbus: 175.6 and NexGen Flex: 183.4; *P* = 0.037. Mean operation time was 53 min for Columbus and 66 min for NexGen Flex; *P* < 0.001. With new streamlined instruments operative time became 60 min for the Columbus; *P* > 0.05. Radiological assessment showed no signs of loosening for both groups. Therefore, the Columbus knee system can be recommended for flexion angles up to 140°.

## 1. Introduction

During the last four decades total knee arthroplasty has been an increasingly successful operative procedure for degenerative as well as arthritic joint disease [1–6]. Excellent clinical and radiological results have been reported for many implant designs [7–9]. 

With an increasing demand of total knee arthroplasty in younger and more active older patients, there is an ongoing development of implant design to allow a higher range of motion. However, an increased range of motion could also preserve activities of daily living in senior patients like gardening and kneeling while praying in a church, as well as sporting activities like bowling. There is in all patients a need to preserve the existent bone stock for potential revision operations. 

The Columbus knee system represents a fixed bearing implant, which can be implanted manually with conventional instrumentation or using the X-ray free OrthoPilot navigation system. It is designed as a standard knee system but differs from other existing designs by one very obvious distinguishing feature: the length of the posterior femoral condyles has been reduced and they have a particularly small radius, to allow deep flexion ankles up to 140°.

The thickness of the femoral implant condyles is not increased compared to standard designs, and there is no additional bone resection. This is a complete different design philosophy compared to other high flexion implants, like the NexGen Full Flex system, which can be named the first system specially designed for high flexion ankles.

Because not every modification of implant design or surgical technique has led to success in clinical practice [10–12], the objective of our study was to analyze the Columbus knee system and to compare the results with a consecutive series of NexGen Full Flex knee arthroplasty, operated by the same surgeon in the same technique (no other variation than implant type itself). 

The analyses were based on the two thesis: (1) there is a clinical difference between the 4-year results of the Columbus knee and a specialist high flex implant; (2) there is no difference between operative time and efficiency between the two evaluated knee systems.

## 2. Methods

A prospective series of one hundred nine total knee arthroplasties of the Columbus knee system (Aesculap AG Tuttlingen, Germany) were performed in one hundred one consecutive patients suffering from primary arthrosis of the knee, operated with manual instrumentation from August, 2004 until March, 2010. The last 11 cases were operated with second-generation instruments (streamlined; Aesculap), which were designed in a more slim way, but otherwise unchanged. The operative approach (see below) remained unchanged.

Mean followup time was 47.3 month (range 15–81). For comparison with other high flex implant designs we used the 4-year results of a group of twenty-two consecutive patients, who received the NexGen (Zimmer, Warsaw, USA) Full Flex implant between August, 2001 and March, 2002 by the same surgeon and who are still in ongoing control in the outpatient department of our clinic. 

All patients were operated in supine position, and all received one shoot i.v. antibiotics 30 min prior to skin incision (1.5 g cefuroxime). Tourniquet was inflated at 350 mm HG prior to skin incision and deflated after bone cement setting, that is, prior to wound closure. We used a midline skin incision, a medial parapatellar approach, and a femur first technique in all patients. Bone cement was in all cases refobacin palacos 40 g, which was used and prepared with 3rd-generation cementing technique. The patella was never resurfaced. At the end of the procedure two intra-articular drainage and subcutaneous drainage were used, while skin was closed by clips. Patients were mobilized on day of surgery by continuous passive motion and one day after surgery out of bed by physiotherapists. Partial weight bearing of 20 kg was recommended for 14 days, full weight afterwards, and rapid range of motion recovery included in the postoperative regime. For thromboprophylaxis all patients received stockings as well as low-molecular heparin subcutaneously once per day for 4 weeks.

Patients were evaluated in our outpatient department prior to surgery, six and 12 weeks following surgery, as well as on a yearly basis following surgery. They were also evaluated in between these appointments, when they came to outpatient clinic for other reasons, like low back pain, fractures, for example, of the radius, and so forth.

Followup assessment was carried out always by the same surgeon using the Knee Society Score (KSS) [13], which was extended regarding the knee score from 100 up to 106 points, because theoretically the NexGen Full Flex system allows flexion up to 155° and the Columbus knee system up to 140° (Original KSS: subtotal values for range of motion = 1 point for 5° range of motion up to a maximum of 25 points for 125° range of motion).

Operative time was evaluated for both implant groups as well as between knees operated by the streamlined instrumentation of the Columbus knee system and the NexGen Full Flex system. 

Radiological assessment was done on a.p. and lateral X-rays done in standing position while weight bearing six weeks and once yearly postoperatively. Special emphasis was laid on controlling for impingement of the dorsal femoral condyles to the dorsal edge of the implant inlay and for lift-off or radiolucent lines of the ventral tibia base plate. 

Statistical analyses were based on the previously named two theses and evaluated by Student's *t*-test (significant *P* < 0.05).

## 3. Results

### 3.1. Demographic Data

Columbus was in 81 knees in 76 women and 28 knees in 25 men; bilateral cases were 5 women and 3 men. 

Mean age was 72.4 years in women (range 47 to 89) and 70.3 years in men (range 50–81).

NexGen Full Flex was in 18 knees in 18 women and 4 knees in 4 men. 

Mean age was 67.8 years in women (range 58–79) and 67 years in men (range 56–77). 

Through a recall system of our outpatient clinic we were able to evaluate 100% of this implant series. 

**Figure 1:**
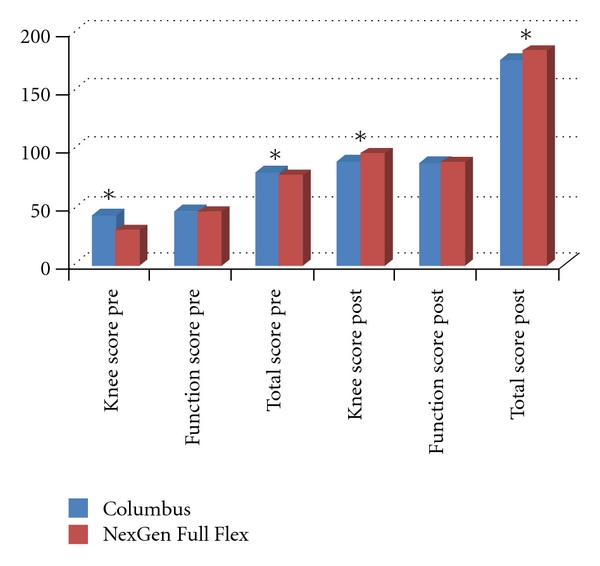
Knee Society Score (KSS) pre- and postoperatively for both the NexGen Full Flex and the Columbus Knee system differentiated in total knee score, knee score, and function score. Through a possible maximum range of flexion up to 155° knee score can reach a subtotal value of 106 points and total score up to 206 points (flexion 5° = 1 point: original score max 25 points, modified score of our study max score 31). *Significant.

Figure 1 gives the total knee, as well as knee and function score for both implant designs pre- and postoperatively. 

Some subtotal values differed for both groups: the difference in the preoperative knee score was significant (lower in the NexGen); *P* < 0.001. The reason for that was a difference of the mean preoperative pain values of the score: Columbus 13.4; NexGen 4.1 (*P* < 0.001), while the function score did not differ significantly.

The preoperative total score difference shown in the figure was significant with *P* = 0.002.

Postoperatively there was no longer a significant difference in the pain values between the groups, but still a significant difference in the subtotal knee score (*P* = 0.004), which was based on a difference in the range of motion (ROM) values: Columbus 21.6 and NexGen 24.3 ≥ *P* < 0.001. This had an effect on the total postoperative knee score: both knee systems showed very good results in favour of the NexGen Full Flex system (*P* = 0.037): Columbus 175.5, NexGen 183.4. 

**Figure 2:**
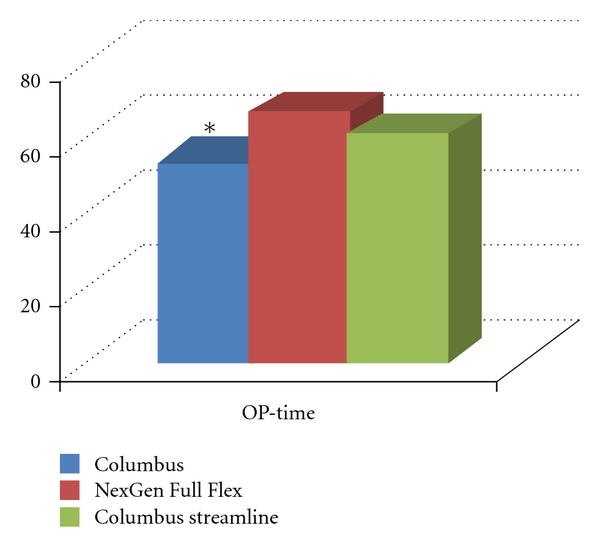
Mean operative time for the Columbus (all operations), the NexGen Full Flex Knee system, and the Columbus streamlined instrumentation [min]. *Significant.

Mean operation time (Figure 2) was 53 min for the Columbus knee and 66 min for the NexGen Full Flex. This was a significant difference with *P* < 0.001. Analyzing a subgroup of 11 cases operated with the streamlined Instruments of the Columbus knee, operative time was 60 min and not significantly different from the NexGen Full Flex system.

There was one revision for postoperative haematoma and one because of traumatic femoral fracture (falling downstairs) in the Columbus group but no other relevant complication (infection, arthrofibrosis, aseptic loosening, or deep vein thrombosis). 

**Figure 3:**
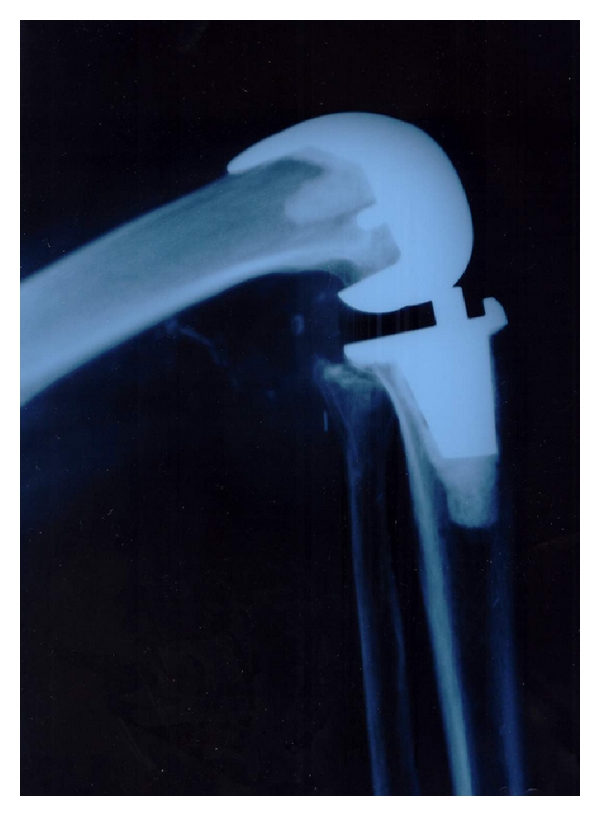
Lateral X-ray of a NexGen Full Flex implant in maximum flexion of the knee joint: patient does not reach the flexion limits of the implant. The dorsal implant condyles are some millimeters longer than the original bone condyles. (Notice the vessel clips in the calf, from earlier surgery).

**Figure 4:**
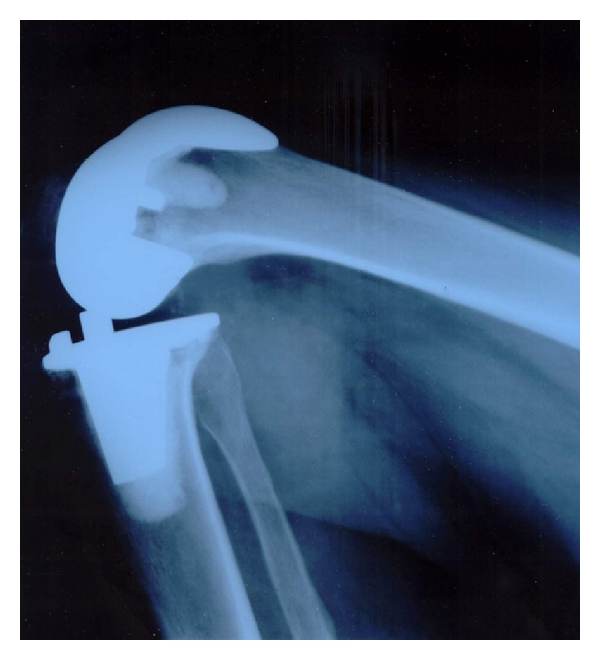
Lateral X-ray of a NexGen Full Flex knee in maximum flexion of the patient: full soft tissue contact of calf and tight. There is no instability or subluxation of the implant. Notice that even in this patient implant range of flexion is not completely used.

**Figure 5:**
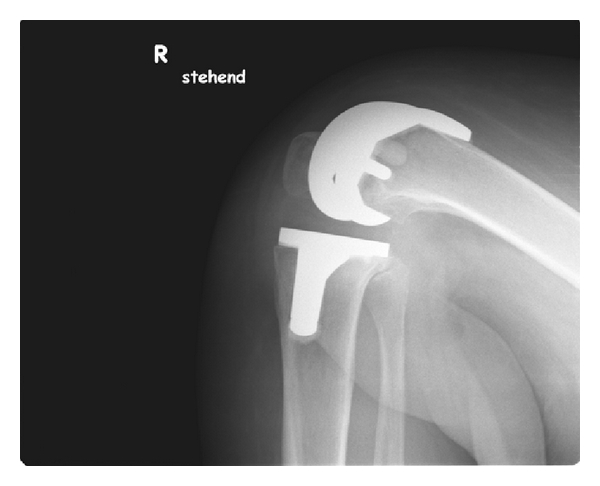
Lateral X-ray of a Columbus knee in maximum flexion: the remaining original posterior condyles do not contact the rim of the polyethylene inlay although there is already full contact of calf and tight. There is no instability or subluxation of the implant.

On radiological assessment we did not see any significant radiolucent line (>2 mm), aseptic loosening, or tilting of the implants. Even in high flexion cases of the Columbus implant we could not detect any “cold flow” or relevant compression between the dorsal parts of the polyethylene inlay to the dorsal femoral condyles. There was no lift-off of the tibial implant. The lateral X-ray views quite frequently showed that the implant-related maximum of deep flexion is not reached up to its limits (Figure 3). Even in patients with very deep flexion ankles, mostly the soft tissue of calf and tight limited the flexion, but not the implant design itself (Figures 4 and 5). 

## 4. Discussion

Although we know that even expensive new developments in total joint arthroplasty design do not guarantee success regarding clinical results and survival rates, most recent studies discuss the operative approach or different types of computer-assisted surgery but do not analyze different implant designs [11, 12, 14–16]. Recent problems regarding short- and medium-term results in total joint arthroplasties have demonstrated the value of clinical research [17].

Clinical results and postoperative Knee Society Score of our Columbus knees as well as for our NexGen group do not stand behind the results reported in the literature for the NexGen system or other implants. They have to be called good to excellent in both groups after 4-year followup time [18–23].

However, we found some significant differences between our two groups: the preoperative total knee score was significantly lower in the NexGen group. Analyzing that difference we found the reason for the lower subtotal knee scores of the NexGen group is based on lower values in the pain score (*P* < 0.001). Retrospectively we had to notice that with good success rates of our total joint arthroplasty operations and increasing demands of the patients, for example, in spare time activities and activities of daily living, ealier regarding limitations of walking, stability and range of motion. This resulted in somewhat higher preoperative knee score values. 

Total knee scores did differ significantly between both groups in favor of the NexGen knee. This was based on a slight difference in favor of the NexGen implant regarding the subtotal mean range of motion values: Columbus 21.6; NexGen 24.3. Looking at every patient's range of motion, we recognized that only one patient with a NexGen full flex implant reached deep flexion angles above 140°, which would be the mechanical flexion limit of the Columbus knee (the patient showed 150° of flexion). 

Roenn et al. [24] stated that there are implants available affecting a higher femoral translation and range of motion [25], while Kim et al. reported that the expected difference between standard knee prosthesis and high flexion implants had not been observed in randomized controlled studies [26]. However, the previously named mean range of motion values correspond to 108° of flexion for the Columbus knee and 121.5° for the NexGen knee. These values are above the flexion angles reported for standard knees [1, 3, 27]. Although the mechanical limits of both implant types are not reached, we believe that this deeper flexion and the known quicker rehabilitation in high-flexion implants [28] are two important reasons for high patients satisfaction in both of our groups.

Achieving deep flexion is influenced by surgical factors, rehabilitation regime, patient factors, and implant design. To exclude bias from these first three parameters, we used a standardized technique for all of them, as it is described previously. This is the main strong point of our study: variation of only one parameter, which is implant design, is able to answer the study objective. 

Although computer navigation is possible for the Columbus knee system, this would have included a second parameter of variation compared to the NexGen group and would have influenced the question of the study [29], especially regarding operative time.

Because manual total knee arthroplasty versus computer-assisted surgery showed similar results for the Columbus knee system [22], we were able to exclude in this study the use of the OrthoPilot navigation system, which we recommend for special cases, when, for example, femoral intramedullary instrumentation is not possible through screws, plates, or other implants from former femoral fractures if patients had already long-stem total hip endoprosthesis—without a disadvantage for the patient. 

One limitation of our study may be the small control group. However, these cases were the only ones in our data pool, when a high flexion knee system was implanted by one of the authors under exactly the same operative setting.

In both groups of our study indication to the operation was similar: osteoarthritis of the knee, without any further option for nonoperative treatment and without bone or ligament defects resulting in a stabilized knee implant. In favor of standardization we accepted this limitation of different group size. 

Surgical time as one parameter of total joint arthroplasty stands well behind functional results. Reduced operation times do not only mean a possible reduced rate of infections, deep vein thrombosis, and so forth but also influence the hospital itself. Theatre time, cleaning instruments, and trays, as well as packing of them, mean hospital costs. And for these parameters there is no general difference between national health systems or hospital trusts. There may only be a difference in the individual amount of them.

The average surgical time in the NexGen group was with 66 min significantly longer than in the Columbus group (=55 min). However, for both groups surgical times are clealry lower than told in the literature [14, 15, 30]. 

The reason for the extra time in the NexGen group could be the additional instruments for the posterior recut of the dorsal femoral condyles in the NexGen system. For termination in the theatre one has to notice that this is not only one additional instrument but a collection of different sizes for this recut guide, pins/screws, and so forth. However, the advantage of the Columbus knee was lost with the new generation of instruments, called streamlined, which has to be used now for implantation. These new instruments do not include additional operative steps or resection guides for the Columbus knee but are different in their design itself. This demonstrates clearly that not only the number but also the design of instrumentation should be well advanced to allow accurate and efficient procedures. Looking at the previously stated surgical times, both knee systems can be recommended regarding that parameter. 

**Figure 6:**
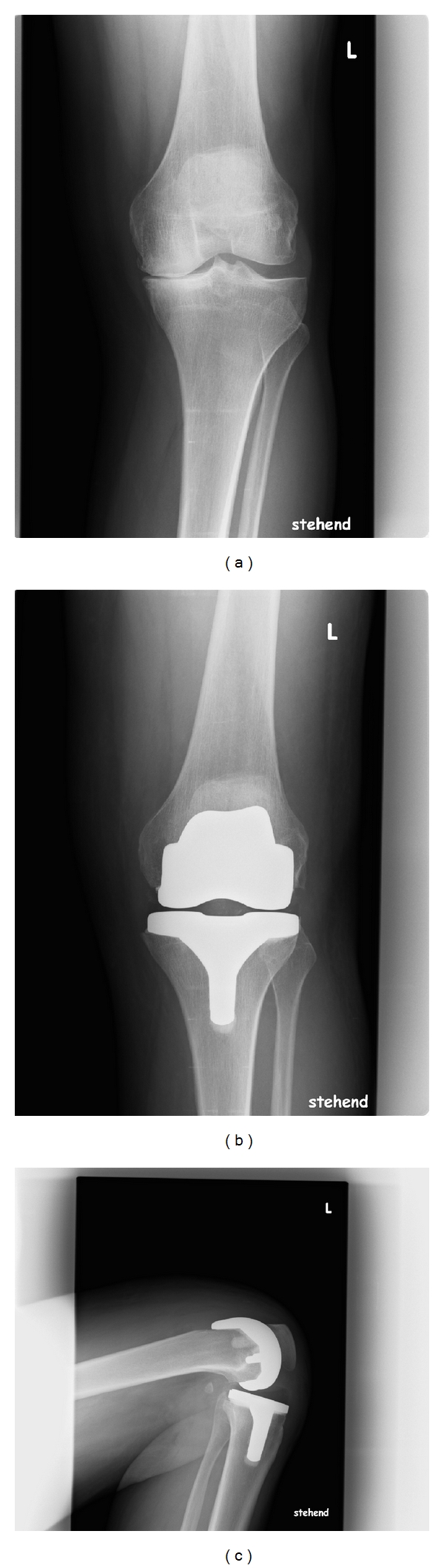
X-ray of a Columbus knee: a.p. view pre- and postoperatively (standing position with full weight bearing) and lateral view in maximum flexion. There is soft tissue contact of calf and tight. The reduced length of the posterior implant condyles led to bone contact of the remaining original posterior condyles with the posterior edge of the polyethylene inlay.

In radiological assessment we did not see any dorsal impingement problems of the remaining bone of the dorsal femoral condyles on the polyethylene inlay in the Columbus knee system. Even in one case when the remaining posterior condyle bone touched the inlay rim (Figure 6), the patient experienced no pain and we did not see any cold flow or inlay destruction. There was also no lift-off of the ventral tibial implant—we would notice by radiolucent line at the cement implant interface ventral to the tibial implant stem—which one would expect in pathological dorsal pressure given from the condyles to the dorsal tibia. 

We like to remark that we do reconstruct the natural individual tibial slope of the patient's tibia, shown on the preoperative lateral X-ray view—in severe destructed knee joints, we use X-ray of the contra lateral side—which is known to be 7° [31]. That means we do in most cases a tibial resection of 4° posterior slope (3° slope included in the implants inlay).

One may ask why not resect the remaining condyle to achieve a nice bony curve from implant to the posterior femoral corticalis: Beside the usual management of posterior capsular release and removal of osteophytes we believe in preserving as much bone as possible (recognize the difference in bone resection in Figures 4 and 5), especially when “plastic surgery of the X-ray” may lead to a weak posterior corticalis. A weak posterior corticalis may lead, similar to ventral cortical notching, to femoral fractures. 

In the NexGen group we did not see any significant radiolucent lines at 4-year followup, which is in contrast to the report of Cho et al. [19]. They found around the femoral component progressive radiolucent lines in 13.8% of their cases and suggested passive maximal flexion activity of their patients as the reason for that. Although our patients have a need for deep flexion in more dynamic free time activities like bowling and gardening with short-term stress on the femoral implant, in contrast to passive stress of longer duration like in squatting and kneeling in other cultures, we should consider very closely that problem during the further followup time. 

## 5. Conclusion

The overall results of our study are encouraging for both systems. The clinical results of the Columbus knee system are comparable with those of the NexGen Full Flex knee system.

Operative time was for both systems under the standardized conditions of similar advanced manual instrumentation. 

We did not see any problems of impingement of the proximal, resected dorsal femoral condyles—uncovered by the Columbus implant—to the polyethylene inlay and no lift-off phenomenon of the tibial implant.

Therefore, the Columbus knee system can be recommended to achieve flexion angles up to 140°.
